# A Case of Aortic Mural Thrombus Presenting as Recurrent Shoulder Pain Discovered by the Presence of Splinter Hemorrhages

**DOI:** 10.7759/cureus.103027

**Published:** 2026-02-05

**Authors:** Abigail Hall, Steven J Laxton, Michael Silberman

**Affiliations:** 1 Emergency Medicine, University of Tennessee Health Science Center, Memphis, USA

**Keywords:** aortic arch thrombus, aortic mobile thrombus, aortic thrombus, claudication, extremity claudication, shoulder pain

## Abstract

Progressive claudication of the upper extremity is uncommon and can be misattributed to cervical radiculopathy or even shoulder pathology, such as tendonitis or bursitis. We report a patient presenting to the emergency department with several months of worsening left upper-extremity pain initially managed as suspected radiculopathy, but, in retrospect, was upper-extremity claudication. This was discovered through the presence of lesions beneath the fingernails of the left hand, splinter hemorrhages. A computed tomographic angiography (CTA) of the chest for aortic and vascular evaluation demonstrated a mural thrombus partially occluding the left subclavian artery. The patient was admitted for further management and observation. Following initiation of systemic anticoagulation, symptoms had significantly improved, and the patient was discharged home in stable condition. This case underscores the diagnostic importance of bedside examination and vascular imaging when evaluating atypical upper-extremity claudication. This also highlights arterial thrombus as a potentially reversible cause of upper-extremity pain when promptly recognized and treated.

## Introduction

Splinter hemorrhages (SHs), first characterized in the early 20th century, are linear, non-blanchable, reddish-brown to black longitudinal streaks that appear beneath the nail plate. They are usually painless but may be associated with tenderness or sharp, burning pain. This physical examination finding is classically associated with acute infective endocarditis; however, SHs are not a sensitive indicator and have been described in a wide range of conditions, including vasculitis, drug reactions, connective tissue disorders, chronic meningococcemia, trauma, exposure to high altitude, and even routine activities of daily living related to manual labor [1]. The pathophysiology of SHs is not well understood but is likely multifactorial and dependent on the underlying disease process. Some sources state they occur due to fragile nail bed capillaries that bleed into the nail bed ridges [2]. In this case, the patient’s SHs are thought to represent microemboli originating from a primary aortic mural thrombus (PAMT) [3]. Recognition of SHs should therefore prompt a thorough history and physical examination and consideration of early vascular imaging when indicated.

PAMT is an uncommon entity occurring in an otherwise normal aorta without aneurysm, significant atherosclerosis, or dissection, with a prior reported incidence of 0.45%. These thrombi can be sessile or pedunculated with a free-floating segment [4]. In addition, free-floating thrombi are associated with an increased risk of peripheral embolization [5]. In a clinical series, the descending thoracic aorta and aortic arch are the most commonly identified sites of mural thrombus formation with the ascending aorta being the least reported as the least affected segement [6]. The true incidence of PAMT remains unknown, in part because it is frequently asymptomatic and may only be detected incidentally on imaging. The pathogenesis of PAMT is heterogeneous and involves factors that increase hypercoagulability, including but not limited to cigarette smoking, malignancy, hormone replacement therapy, steroid use, heparin-induced thromboembolism, inflammatory disorders, and primary endothelial abnormalities [7]. Clinical presentation varies with thrombus location and may include acute myocardial infarction and cerebrovascular accident when the thrombus is located in the ascending aorta, as well as limb ischemia, bowel ischemia, and visceral arterial embolism with more distal lesions. There are no established evidence-based guidelines for the management of PAMT owing to its rarity. Systemic anticoagulation is generally considered first-line therapy, while surgical or endovascular intervention is often considered on a case-by-case basis [7].

## Case presentation

In August 2025, a 55-year-old male presented to the emergency department with new-onset nail changes and intermittent cyanosis of his fingers, including black discoloration at the tip of his left second digit. He reported intermittent but progressively worsening paresthesia and pain in the left hand and fingers over the previous four to five weeks. Prior to presentation, he had been under the care of an orthopedic specialist for presumed cervical radiculopathy. Plain radiographs of the shoulder, arm, and hand were reportedly unremarkable, and he was treated empirically with injected corticosteroids, which provided no significant relief. 

He sought emergency care after his symptoms recurred, notably with black discoloration of the left second fingertip. On physical examination, SHs were noted in multiple fingernails of the left hand, along with ischemic changes involving the distal phalanx of the second digit. Cardiovascular exam was unremarkable, and no murmurs were appreciated. 

**Figure 1:**
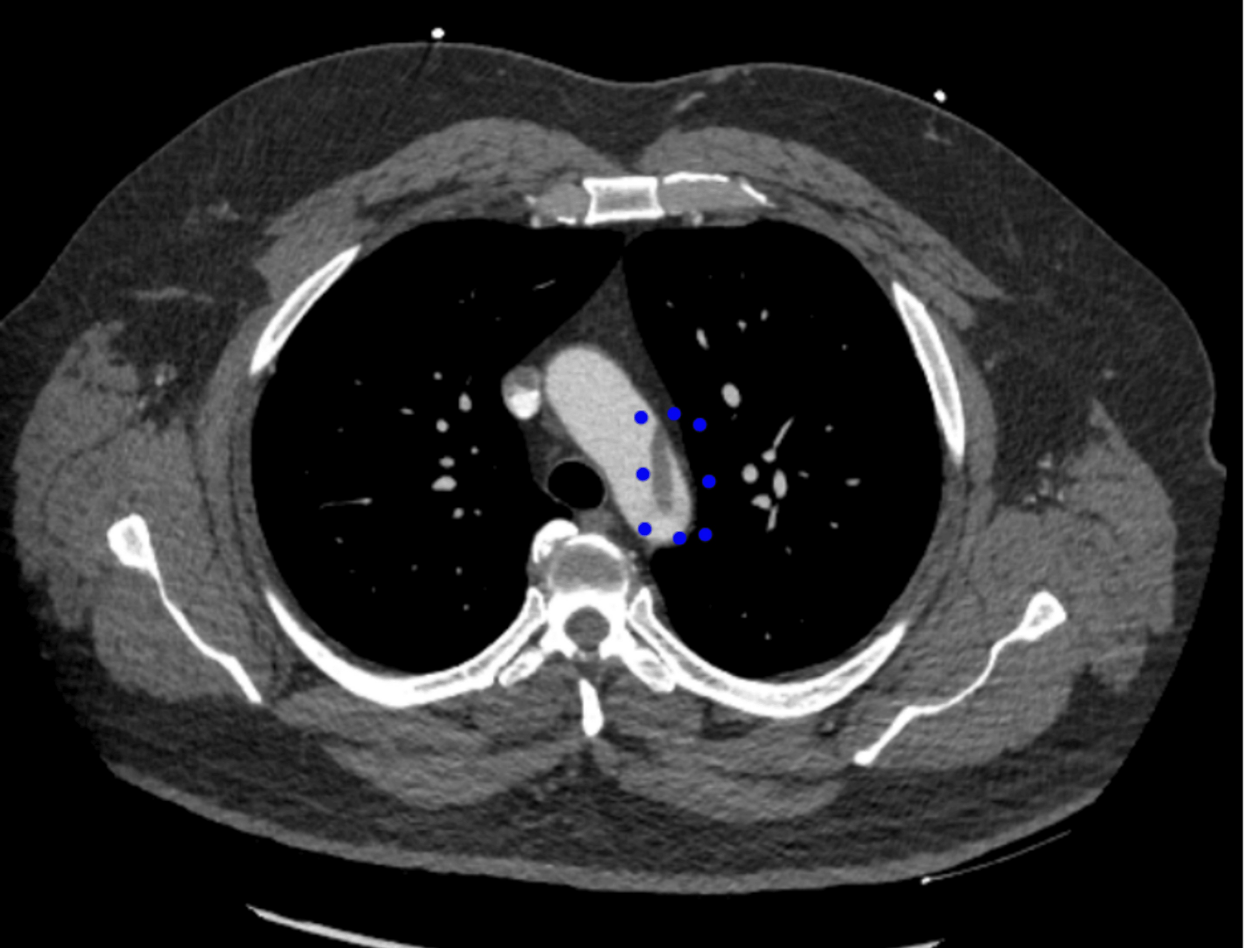
CT scan of the chest demonstrating the aortic mural thrombus This image shows the CT scan of the chest with IV contrast that was taken highlight the mural thrombus noted in the arch of the aorta with blue dots.

Given the concern for vascular phenomena, a CT (computed tomography) angiogram of the chest was performed, which revealed a mural thrombus in the distal aortic arch (Figure 1) extending into the proximal descending thoracic aorta, with a thrombus extension partially occluding the left subclavian artery. Vascular surgery was consulted, and anticoagulation with intravenous heparin at a dose of 18 units/kg/hr was initiated. The patient was admitted to the hospital for observation and had improvement in exam findings. 

After multidisciplinary evaluation, vascular surgery recommended discharge on oral anticoagulation (apixaban), aspirin, and a high-intensity statin (rosuvastatin 20 mg daily), with outpatient follow-up with vascular surgery. The patient was discharged on hospital day two in stable condition and had no further sequelae of the aortic thrombus on follow-up with vascular surgery. 

## Discussion

This case highlights the critical role of thorough physical examination in identifying systemic disease processes that may otherwise be overlooked. One study found that a complete history and physical alone can establish the diagnosis in up to 61% of cases, even before laboratory testing or advanced imaging are obtained [8]. The patient initially presented to the emergency department with progressive claudication of the left upper extremity, including pain and paresthesias that were previously attributed to cervical radiculopathy. However, the progression of symptoms, particularly the distal digit ischemia and nail bed findings, warranted a reevaluation of the diagnosis.

SHs, a nonspecific but important physical examination finding, can serve as a subtle yet vital clinical clue when interpreted in the appropriate clinical context. In this case, their identification, along with signs of digital ischemia, prompted a broader vascular workup including a CT angiogram of the chest. While classically linked to infective endocarditis, SHs may also result from embolic phenomena originating from non-valvular sources, such as aortic mural thrombi (Haber, Schwiebert). The absence of a murmur on exam and a normal orthopedic and neurologic exam helped direct focus away from endocarditis and neurologic or musculoskeletal causes toward a more systemic vascular process.

Primary mural thrombus of the thoracic aorta is a rare but serious condition that can lead to arterial and/or venous embolization, particularly in the absence of aneurysmal disease or overt atherosclerosis. There is often a delay in diagnosis due to its initially asymptomatic nature, nonspecific symptoms, and low index of suspicion. Often, the initial presentation of PAMT is peripheral embolism rather than symptoms referable to the aorta itself [9]. In this case, a physical finding as subtle as SHs, coupled with ischemic changes in a digit and a careful re-evaluation of the clinical picture, was instrumental in revealing the diagnosis of free-floating aortic thrombus, a condition with significant thromboembolic risk to life and limb. Management typically includes systemic anticoagulation, and in some cases, surgical or endovascular intervention [10]. This patient responded well to medical therapy and was safely discharged with close follow-up.

Ultimately, this case serves as a reminder that a thorough physical exam is advantageous and can redirect clinical reasoning and profoundly impact patient outcomes. A careful, hands-on evaluation remains a cornerstone of effective diagnostic medicine, particularly in an era increasingly reliant on imaging and subspecialty referrals [11].

## Conclusions

This case illustrates how the subtle physical examination finding of SHs, combined with progressive, atypical upper-extremity claudication, can uncover a rare but clinically significant diagnosis of PAMT.

Early vascular imaging and prompt multidisciplinary involvement facilitated timely diagnosis and initiation of systemic anticoagulation, resulting in symptom improvement and prevention of further thromboembolic complications. Clinicians should maintain a high index of suspicion when evaluating unexplained limb ischemia, especially when accompanied by nail bed changes, and consider aortic pathology among the differential diagnoses even in patients without aneurysm or advanced atherosclerosis. This case reinforces the enduring value of a meticulous bedside examination as a catalyst for appropriate imaging, accurate diagnosis, and optimized patient outcomes.
